# One-Step Green Hydrothermal Synthesis of Few-Layer Graphene Oxide from Humic Acid

**DOI:** 10.3390/nano8040215

**Published:** 2018-04-03

**Authors:** Guangxu Huang, Weiwei Kang, Qianhao Geng, Baolin Xing, Quanrun Liu, Jianbo Jia, Chuanxiang Zhang

**Affiliations:** 1 College of Chemistry and Chemical Engineering, Henan Polytechnic University, Jiaozuo 454003, China; guangxu1369@163.com (G.H.); BlackPanther1010@163.com (Q.G.); baolinxing@hpu.edu.cn (B.X.); jiajianbo@hpu.edu.cn (J.J.); 2 Collaborative Innovation Center of Coal Work Safety of Henan Province, Jiaozuo 454003, China; 3 Henan Key Laboratory of Coal Green Conversion, Jiaozuo 454003, China; 4 School of Chemistry and Chemical Engineering, Southeast University, Nanjing 211189, China; weiweikangwell@163.com; 5 Henan Province Industrial Technology Research Institute of Resources and Materials, Zhengzhou University, Zhengzhou 450001, China

**Keywords:** graphene oxide, hydrothermal treatment, hydrolysis, humic acid, supercapacitor

## Abstract

The conventional synthesis route of graphene oxide (GO^G^), based on Hummers method, suffers from explosion risk, environmental concerns and a tedious synthesis process, which increases production costs and hinders its practical applications. Herein, we report a novel strategy for preparing few-layer graphene oxide (GO^H^) from humic acid via simple hydrothermal treatment. The formation of GO^H^ is mainly attributed to the hydrolysis, oxidation and aromatization of humic acid under hydrothermal conditions. The as-prepared few-layer GO^H^ has typical morphology (thin and crumpled sheets with the thickness of ~3.2 nm), crystal structure (a Raman I_D_/I_G_ ratio of 1.09) and chemical composition (an X-ray Photoelectron Spectroscopy (XPS) O/C atomic ratio of 0.36) of few-layer GO^G^. The thermally reduced GO^H^ (r-GO^H^) delivers considerable area capacitance of 28 µF·cm^−2^, high rate capability and low electrochemical resistance as supercapacitor electrodes. The described hydrothermal process shows great promise for the cheap, green and efficient synthesis of few-layer graphene oxide for advanced applications.

## 1. Introduction

Graphene, a single sheet of sp^2^-bonded carbons arranged in a honeycomb lattice, has attracted great attention because of its remarkable electrical [1], mechanical [2] and thermal properties [3]. Large-area films and small sheets are two major forms of graphene used for various applications [4]. Chemical vapor deposition (CVD) is an efficient method to produce large-area high-quality graphene films [5] and small graphene sheets are typically made by thermal or chemical reduction of GO^G^ [6]. GO^G^ is not only the key intermediate for production of graphene-based materials [7] but also very useful for functional materials such as electromagnetic wave absorption material [8], gas sensor [9], lithium-ion battery anode [10], supercapacitor electrode [11] and catalyst [12]. Generally, GO^G^ is prepared by the ultrasonic exfoliation of graphite oxide, which can be obtained from graphite powders by harsh oxidation according to Hummers method, using oxidants such as KMnO_4_ dissolved in concentrated H_2_SO_4_. Unfortunately, this strategy is plagued by explosion risk, environmental concerns and tedious synthesis process.

Graphite oxide is a lamellar solid with sp^2^/sp^3^-hybridized carbon domains, containing epoxide and hydroxyl groups on the basal planes with carbonyl and carboxyl groups at the edges [13] that make graphite oxide water-soluble. Humic acid (HA) and Fulvic acid (FA) are two kinds of natural polymer materials, consisting of a skeleton of alkyl/aromatic units cross-linked by oxygen-containing functional groups such as carboxylic, hydroxyl, ketone and Quinone groups [14]. Thus, both HA and FA are considered as readily available, low cost source of graphite-oxide-like materials [15]. However, HA molecules adopt coiled and compact structure in water, forming aggregation and ultimately precipitation [16], which is primarily due to the relatively stronger interactions among hydrophobic portions and lower oxygen content compared to the graphite oxide. Moreover, besides aromatic rings, the huge structure of HA molecule contains aliphatic chains which hardly exists in graphite oxide. In this sense, after a given oxidation with maintaining or increasing sp^2^ carbon content, the humic acid will be converted to GO^H^. Unfortunately, oxidative treatment of carbon-based materials generally gives rise to decreased sp^2^ carbon content, which is the case for GO^G^ preparation from graphite. As for FA, it is water-soluble because of higher oxygen content and lower molecular weight compared with HA.

Hydrothermal treatment is a thermo-chemical conversion technique, resulting in efficient hydrolysis, pyrolysis, dehydration, polymerization and aromatization of organic precursors and bestowing the products with high oxygenated functional group content as well as condensed aromatic structures [17,18,19,20]. Biomass and GO^G^ can be converted to solid char and graphene using hydrothermal treatment method, respectively, which essentially involve the carbonization and reduction process [19,21]. Small molecular soluble byproducts such as aromatic compounds, polysaccharide, aldehydic, ketonic and furan derivatives also formed during hydrothermal treatment of biomass, from which solid products generated by further polymerization [17,18]. Water-soluble and low-molecular weight FA can be converted into graphene quantum dots based on the pyrolysis, self-assemble and dehydration [17]. However, to the best of our knowledge, counterpart studies on water-insoluble and high molecular weight HA and the corresponding reaction mechanism have not been reported yet.

Here we report a green, cheap and efficient strategy for the preparation of few-layer GO^H^ via simple hydrothermal synthesis using HA as starting material, which is expected to be a promising alternative of the Hummer’s method. Under hydrothermal conditions, insoluble HA was partially “dissolved” in water and the GO^H^ solution but not small molecular product was obtained simultaneously. The morphology, crystal structure and chemical composition of the as-prepared GO^H^ were characterized in detail.

## 2. Materials and Methods

### 2.1. GO^H^ Synthesis

**Figure 1:**
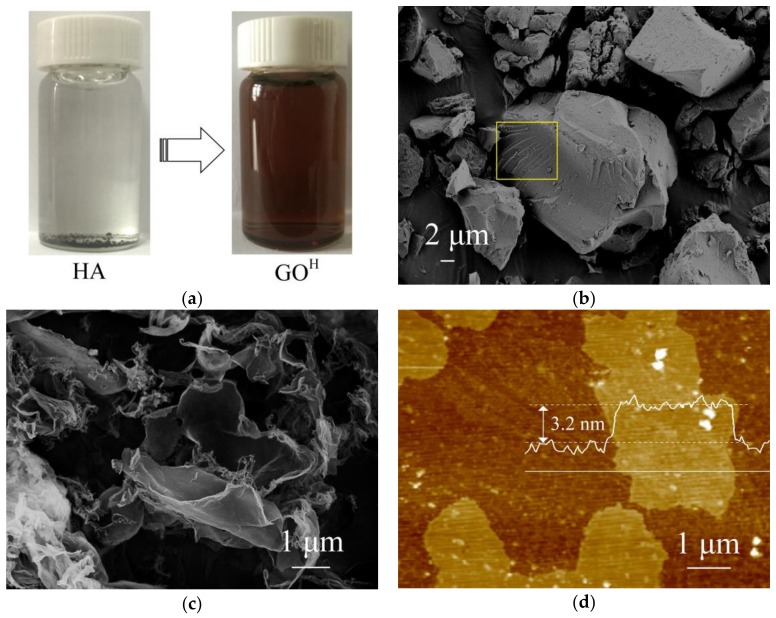
(**a**) A photograph of humic acid (HA) and as-prepared Graphene Oxide (GO^H^) solution; (**b**) Scanning electron microscopy (SEM) image of HA; (**c**) SEM of GO^H^; (**d**) Atomic Force Microscopy (AFM) of GO^H^.

Typically, HA solid particles (Linhai Humic acid Co., Linhai, China) were added to the deionized water, which sank to the container bottom rapidly (the left in Figure 1a) because of its insolubility. The solid-liquid mixture of HA and deionized water was loaded into a Teflon-lined stainless-steel auto-clave and underwent a hydrothermal process at the temperature of 190 °C for 10 h. The resultant homogeneous mixture was sonicated for 30 min, centrifuged (9000 rpm) for 10 min to remove insoluble byproducts and the brown GO^H^ solution was obtained (the right in Figure 1a). The GO^H^ solid can be collected by freeze-drying process and the reduced GO^H^ (r-GO^H^) was further prepared from GO^H^ by thermal reduction at 900 °C for 40 s under N_2_ atmosphere.

### 2.2. Characterization

Scanning electron microscope images were acquired with a field emission scanning electron microscope (FESEM, JEOL, JSM-6390LV, Tokyo, Japan). Atomic force microscopy (AFM, Bruker, Dimension Edge, Karlsruhe, Bade, Germany) was used to determine the thickness of the GO^H^. Transmission electron microscopy (TEM) measurements were performed using a JEOL JEM-2100 instrument (Tokyo, Japan) operated at 200 KV. X-ray diffraction (XRD) analyses were carried out by an X-ray diffractometer (Smart-Lab, Rigaku, Tokyo, Japan) using monochromatic Cu Kα1 radiation at 40 kV. The Raman spectra were measured using a Renishaw inVia Raman spectrometer (London, UK) with a 520 nm excitation argon laser. Ultraviolet–visible spectra were obtained using a Varian Cary 300 Bio UV-visible spectrophotometer (Santa Clara, CA, USA). XPS analyses were carried out on an Axis Ultra electron system (Waltham，MA;USA) using Mg/Al X-ray source at 225 W. Fourier transformation infrared (FTIR) spectra were measured in KBr pellets on a Bruker TENSOR27 spectrometer (Berlin, Germany). Solid-state ^13^C nuclear magnetic resonance (NMR) spectra were recorded on a Bruker (Avance III) instrument (Fu tengburg state, Germany) operating at 400 MHz. Brunauer-Emmett-Teller(BET) surface area and pore structure measurements were conducted using a Quantachrome Autosorb-iQ-MP analyzer (Boynton Beach, FL, USA) at 77 K.

The electrodes were fabricated by pressing a homogeneous mixture of r-GO^H^ (85 wt %), black carbon (10 wt %) and polytetrafluoroethylene (5 wt %) under the pressure of 10 MPa. The electrochemical properties were measured using two-electrode cells with 3 M KOH solution as the electrolyte. The galvanostatic charge/discharge (GC) and cyclic voltammetry (CV) were carried out by an electrochemical analyzer system (SCTS, Arbin, SCTS, Arbin, College Station, TX, USA). The area capacitance of a single electrode was calculated from the discharge part of GC curves according to the formula *C* = (2000 × *I* × Δ*t*)/(*S* × Δ*V*), where C is the area capacitance in µF·cm^−2^, I is the discharge current in A, S the surface area of the active materials in cm^2^ and Δ*t*/Δ*V* is calculated from the slope of the discharge curve. The electrochemical impedance spectroscopy (EIS) was conducted on an electrochemical test system (Parstat2273, Princeton, NJ, USA).

## 3. Results and Discussion

### 3.1. Characterizations of GO^H^

The morphology of the GO^H^ sheets and HA precursor was investigated by scanning electron microscopy (SEM) and atomic force microscopy (AFM). As shown in Figure 1b, the HA raw material exhibits angular blocky texture stacked with thin layers (see yellow rectangle in Figure 1b), suggesting a regular arrangement resembling a crystal lattice structure. It is surprising that these HA blocks were fully exfoliated after simple hydrothermal process. Interestingly, the resultant GO^H^ material demonstrates randomly crumpled sheets with good flexible and ultrathin nature (Figure 1c), similar to that for GO^G^ [9]. It is observed that the lateral dimensions of the GO^H^ sheets are higher than 3 μm. A histogram of thickness acquired across GO^H^ film using AFM shows a mean thickness of about 3.2 nm (Figure 1d), confirming the few layers feature (≤3 layers) and the presence of oxygen containing functional groups on the basal plane [22,23].

**Figure 2:**
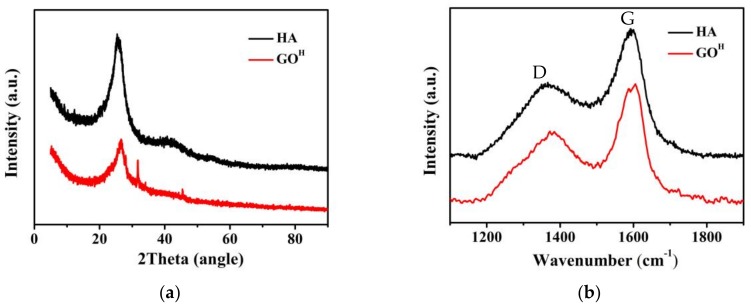
(**a**) X-Ray Diffraction (XRD) patterns; (**b**) Raman spectra of HA and GO^H^.

Figure 2a shows the typical XRD patterns of HA and GO^H^. It can be seen that the HA exhibits strong (0 0 2) diffraction peak at ~26°, indicating the π−π stacking of graphitic carbons [24], which is consistent with the SEM results. By contrast, GO^H^ shows relatively weak (0 0 2) diffraction peak, suggesting the few atomic layers structure of GO^H^. Raman spectroscopy was used to further check the crystalline quality of the HA flakes and GO^H^ by monitoring the relative intensity of the D peak (associated with sp^3^ carbon atoms or defects, at ~1370 cm^−1^) and the G peak (revealing sp^2^ plane of graphene sheets, at ~1595 cm^−1^). The GO^H^ demonstrates an I_D_/I_G_ ratio of 1.09 (Figure 2b), which is similar to that of the GO^G^ [25]. It is worth mentioning that the GO^H^ possesses a lower I_D_/I_G_ value than its counterpart (1.28), suggesting more sp^2^ hybridized carbon domains, less lattice distortions and defects. It can be concluded that non-aromatic components within the humic acid can self-assemble into graphitic hexagonal matrix under hydrothermal conditions, resulting in an increased sp^2^ carbon content of GO^H^. The UV-vis absorption spectrum of GO^H^ solution presents a strong absorption peak at 230 nm (Figure S1), which is related to the π−π* transition of aromatic sp^2^ domains [26].

**Figure 3:**
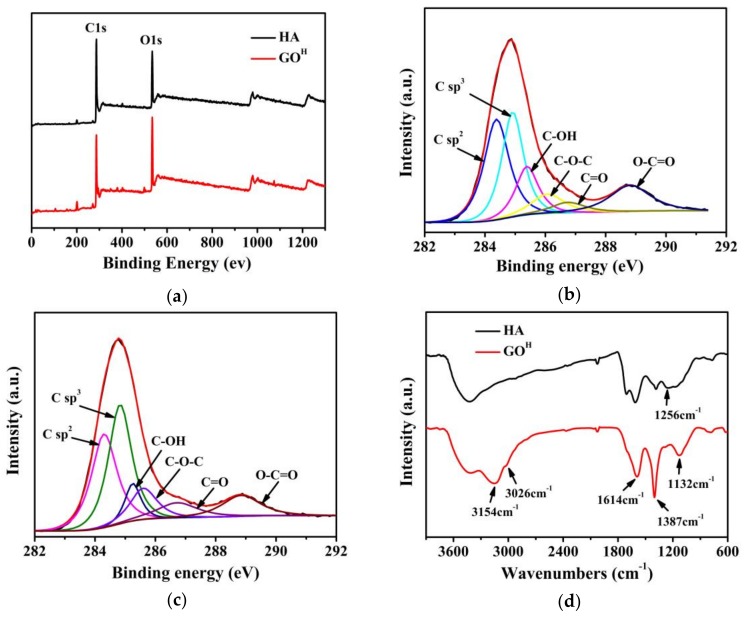

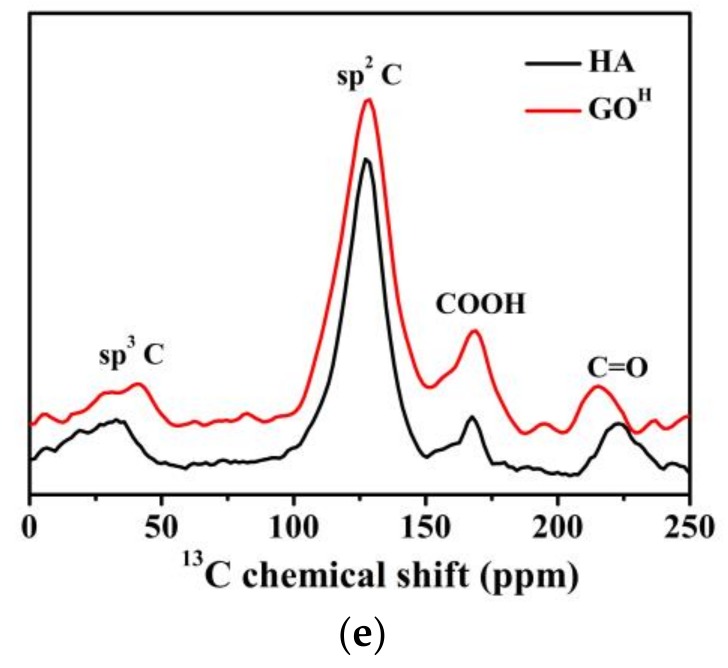
(**a**) Full-range XPS spectra of HA and GO^H^; (**b**) C1s XPS spectra of GO^H^; (**c**) C1s XPS spectra of HA; (**d**) FTIR and (**e**) ^13^C NMR of HA and GO^H^.

The XPS survey spectra shows C1s peaks at ca. 285.7 eV along with O1s peaks at ca. 532.6 eV (Figure 3a), indicating that GO^H^ has a lower C/O atomic ratio (2.76) than HA (3.53). The deconvoluted C1s peak of GO^H^ discloses the presence of 32.0 atom % of sp^2^ C=C (284.3 eV), 28.7 atom % of sp^3^ C−H/C−C (284.9 eV), 13.8 atom % of C−OH (285.3 eV), 7.8 atom % of C−O−C (285.8eV), 4.4 atom % of C=O (286.7 eV) and13.4 atom % of C(O)−O (288.8 eV) groups (Figure 3b) [27]. It is worth noting that, compared to GO^H^, the HA possesses lower content of C=C (29.9%), C−OH (7.5%) and C(O)−O (10.0%), whereas higher content of C−H/C−C (34.6%), C−O−C (10.5%) and C=O (7.6%) (Figure 3c). The FTIR spectra further identify the changes of functional groups before and after the hydrothermal processing of HA (Figure 3d). Compared with HA, the GO^H^ shows a stronger aromatic C=C peak from oxidized sp^2^ bonds at 1614 cm^−1^ and tertiary C−OH peak at 1387 cm^−1^, new peaks including phenolic−OH at 1132 cm^−1^, the −OH of −COOH at 3154cm^−1^ and sp^2^ hybridized carbon networks of 3026 cm^−1^ (shoulder peak). However, the peak at 1256 cm^−1^ related to C−O stretching of aryl ethers and epoxy symmetric rings disappeared [28]. The ^13^C NMR spectra of HA and GO^H^ are shown in Figure 3e. Compared to HA, the GO^H^ exhibits higher percentages of aromatic carbons (~130 ppm) and −COOH groups (~168 ppm) related to total carbon, while lower aliphatic carbons (~35 ppm) and C=O groups (~218 ppm).

According to the XRD, Raman, XPS, FTIR and NMR results, the reaction process for the formation of GO^H^ from HA can be summarized as follows. Some sp^3^ C−H/C−C were converted into sp^2^ C=C, some aryl ethers were hydrolyzed to produce phenol hydroxy groups [29,30]. The carboxyl content increased (the pH value of reaction mixture decreased from 4.6 to 3.9 accordingly), some of which may be converted from C=O groups. The HA underwent an oxidation process, resulting in a higher oxygen content of the GO^H^ than that of HA, the abundant oxygen containing groups (especially –OH and –COOH) in GO^H^ make it soluble in water. In brief, the formation mechanism of GO^H^ is mainly based on the hydrolysis, oxidation and aromatization of HA during hydrothermal treatment. It is interesting to note that HA underwent oxidation and aromatization process simultaneously under hydrothermal conditions, both of which are beneficial to the formation of GO^H^. While in terms of GO^G^ preparation process based on Hummers method, the graphite is oxidized meanwhile the sp^2^ carbon content decreases.

### 3.2. Characterizations of r-GO^H^

**Figure 4:**
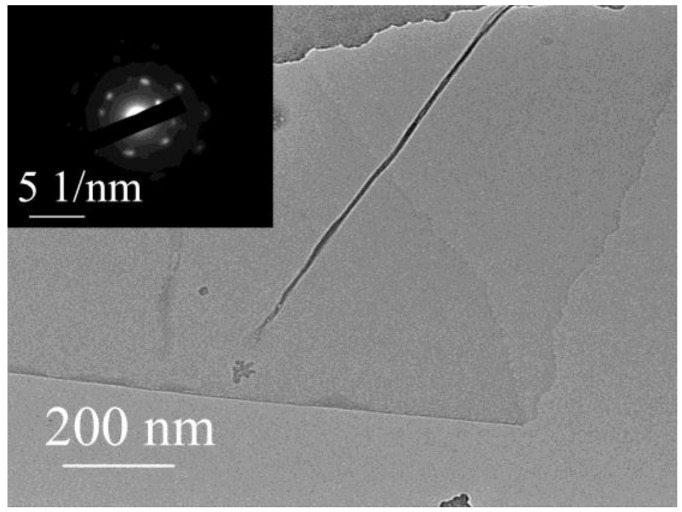
TEM image (EDS of inset) of r-GO^H^.

For practical applications, r-GO^H^ was obtained by the reduction of GO^H^. The transmission electron microscopy (TEM) image demonstrates thin and transparent films (Figure 4a), which is consistent with that produced by liquid-phase exfoliation of graphite [31]. The selected area electron diffraction (SAED) pattern (inset in Figure 4a) exhibits a 6-fold symmetric diffraction, indicating the typical hexagonal crystalline structure of few-layer graphene. The Raman spectrum of the r-GO^H^ demonstrates an I_D_/I_G_ intensity ratio of 1.23 (Figure S2a), which is similar to that of r-GO prepared by classical method [22]. The I_D_/I_G_ value of r-GO^H^ increased in comparison to that of GO^H^ (1.09) since the new graphitic domains are smaller in size to the ones present in GO^H^ [23]. The nitrogen adsorption isotherm of r-GO^H^ (Figure S2b) shows type II isotherm characteristics. The r-GO^H^ exhibits a mesoporous structure with a pore size in the range of 2.5 to 22 nm (Figure S2c), which is consistent with that of the curved graphene sheets reported previously [32]. The r-GO^H^ possesses relatively low BET surface area of 239 m^2^·g^−1^ and total pore volume of 0.26 cm^3^·g^−1^ due to the potential aggregation of r-GO^H^ sheets.

**Figure 5:**
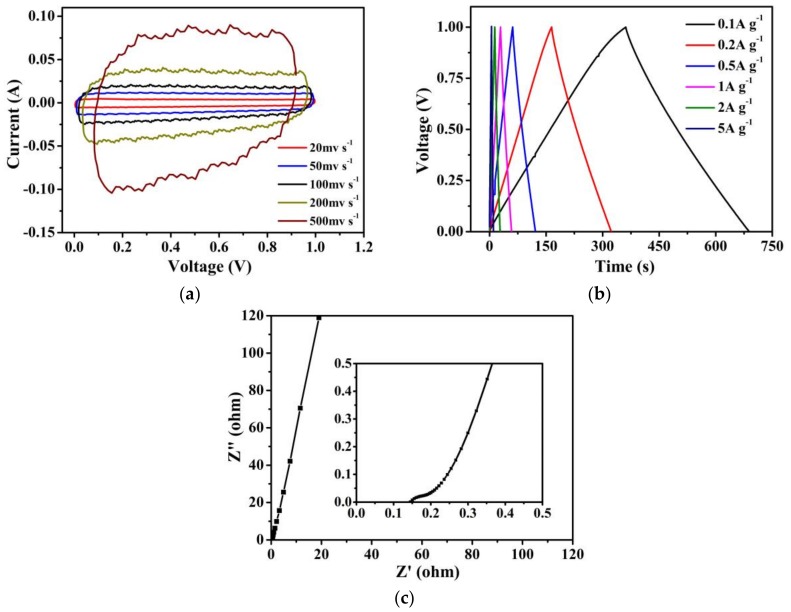
(**a**) CV profiles; (**b**) GC curves; and (**c**) Nyquist plot of r-GO^H^.

The CV profiles of r-GO^H^ exhibits nearly rectangular shapes even at a high scan rate of 500 mV·s^−1^ (Figure 5a), indicative of the typical double-layer capacitive behavior and high rate capability. The GC curves at different current densities have triangular shapes (Figure 5b), meaning good reversible charging-discharging characteristics as supercapacitor electrodes. The area capacitance of the r-GO^H^ electrode is 28 µF·cm^−2^ at a current density of 0.1 A·g^−1^, comparable to or even higher than that of highly functionalized activated carbons [33], nanoporous carbons [34] and porous 3D few-layer graphene-like carbon [35]. High capacitance retention of 76.2% is obtained for a 100-time increase in charging current density from 0.1 A·g^−1^ to 10 A·g^−1^, which agrees with the results of CV. EIS plot (Figure 5c) exhibits a closed 90° slope at a high frequency and a near-vertical line intersection with the real axis at low frequency, indicating a pronounced capacitive behavior and fast ion diffusion. Moreover, the r-GO^H^ shows a low equivalent series resistance of 0.15 Ω (inset in Figure 5c). Meanwhile, the equivalent series resistance of r-GO^H^ is lower than those of reduced graphene oxide nanosheets prepared from humic acid by preliminary carbonization coupled with oxidation-exfoliation-thermal reduction (0.17 Ω) [36], 3D nitrogen-doped activated graphene-like nanosheets (0.38 Ω) [37] and edge-nitrogenized graphene nanoplatelets (up to 0.35 Ω) [38]. The satisfactory electrochemical performances of r-GO^H^ are ascribed to its developed mesoporous structure and good electrical conductivity.

## 4. Conclusions

In summary, HA can be dissolved in water to form GO^H^ after undergoing hydrolysis, oxidation and aromatization processes under hydrothermal conditions. The as-obtained GO^H^ has similar morphology, crystalline structure and composition to GO^G^ prepared from natural graphite by conventional method. The r-GO^H^ delivers considerable area capacitance, high rate capability and good electrical conductivity. This work may propose a low-cost, efficient and environmentally friendly production of GO^H^ for advanced application.

## Supplementary Materials

The following are available online at http://www.mdpi.com/2079-4991/8/4/215/s1.
