# Supplementary material for: One-Step Green Hydrothermal Synthesis of Few-Layer Graphene Oxide from Humic Acid
Source: Nanomaterials (Basel). 2018 Apr 3;8(4):215. doi: 10.3390/nano8040215 (PMC5923545; doi:10.3390/nano8040215)
Supplement: Supplementary file 1 [file nanomaterials-08-00215-s001.pdf]

nanomaterials

One-Step Green Hydrothermal Synthesis of Few-Layer Graphene Oxide from Humic Acid

Guangxu Huang, Weiwei Kang, Qianhao Geng, Baolin Xing, Quanrun Liu, Jianbo Jia and Chuanxiang Zhang

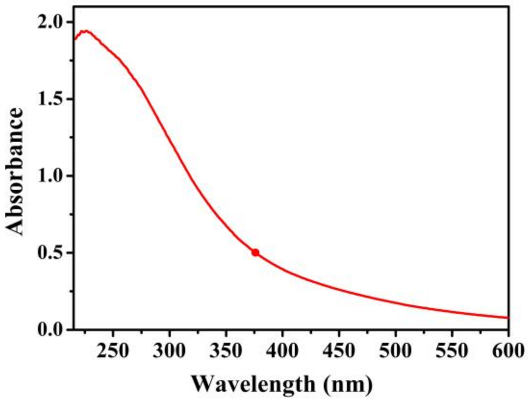

Figure S1. UV-Vis spectrum of GO^H.

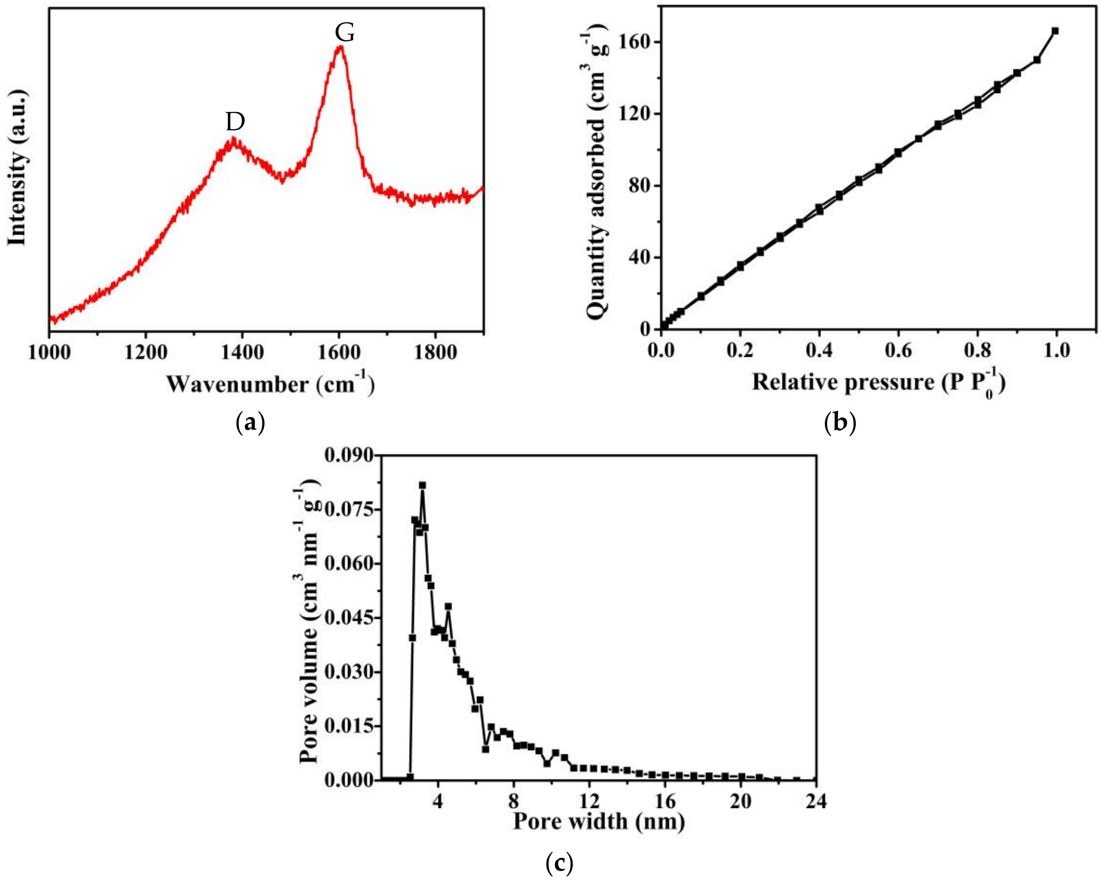

Figure S2. (**a**) Raman spectra, (**b**) N_2 adsorption-desorption isotherm and (**c**) PSD curve of r-GO^H.
